# The impact of body mass index on the efficacy of CDK4/6 inhibitors in patients with metastatic breast cancer

**DOI:** 10.1080/07853890.2025.2597068

**Published:** 2025-12-04

**Authors:** Yiwen Ma, Sijia Wu, Xiaorui Li, Yujun Jiang, Liping Xiao, Ying Wang, Tao Sun

**Affiliations:** Department of oncology, Cancer Hospital of Dalian University of Technology, Cancer Hospital of China Medical University, Liaoning Cancer Hospital and Institute, Shenyang, China

**Keywords:** Body mass index, breast cancer, CDK4/6 inhibitors, hormone receptor-positive

## Abstract

**Purpose:**

Although Body Mass Index (BMI) has been reported to influence breast cancer prognosis, recent evidence challenges the traditional conclusion that high BMI consistently predicts poor prognosis. The aim of this study was to determine the impact of BMI on the efficacy of CDK4/6 inhibitors in patients with metastatic hormone receptor (HR)-positive breast cancer (BC).

**Methods:**

We performed a retrospective chart review of all female patients with metastatic HR-positive BC on a CDK4/6 inhibitor in first- or late-line settings and seen at our academic institution between 2016 and 2023. The primary endpoint was Overall Survival (OS), defined as the time from the start of CDK4/6 inhibitors to death due to any cause.

**Results:**

We identified 212 patients who had received a CDK4/6 inhibitor in the first- or second-line settings for metastatic HR-positive BC. Of the 212 patients, 53.3% (113) had a low BMI (defined as < 24 kg/m^2^) and 46.7% (99) had a high BMI (≥ 24 kg/m^2^). In the first-line setting, mean OS was 46.6 months (95% CI, 38.9 to 54.3) in the BMI-low group versus 78.9 months (95% CI, 69.2 to 88.5) in the BMI-high group (*p* = 0.047). The median PFS was 25 months (95% CI, 19.7 to 30.3) in the BMI-low group versus 33 months (95% CI, 21.4 to 44.6) in the BMI-high group, but the difference between the two groups was not statistically significant (*p* = 0.488). No statistically significant differences were observed in OS and PFS between the two groups in late-line settings (*p* = 0.83; *p* = 0.84).

**Conclusion:**

In HR positive advanced breast cancer patients treated with first-line CDK4/6 inhibitors, lower BMI is associated with poorer prognosis.

## Introduction

Hormone receptor–positive breast cancer, which accounts for approximately 70% of all breast cancer cases, is the most prevalent subtype. HR-positive breast cancer typically exhibits a more favourable prognosis than other subtypes and demonstrates responsiveness to endocrine therapy. However, as the disease progresses, tumours may develop resistance to endocrine therapy. Studies indicated that overactivation of cyclin-dependent kinases CDK4 and CDK6 was one of the primary reasons for endocrine resistance [1]. In recent years, various CDK4/6 inhibitor molecules, including Palbociclib, Ribociclib, Abemaciclib, and Dalpiciclib, have been developed and introduced [2]. The large-scale phase-III clinical trials conducted with the PALOMA, MONALEESA, MONARCH, and DAWNA series have confirmed that CDK4/6 inhibitors play a pivotal role in extending progression-free survival and overall survival in HR-positive breast cancer patients [3].

According to the latest statistics from the World Health Organization, the global rates of overweight and obesity continue to rise, facing a crisis of significantly increased disease burden associated with obesity [4]. Body Mass Index (BMI), as an indicator of obesity, was linked to an increased risk of various cancers. Most studies suggest that BMI-affected breast cancer prognosis, but clinical research conclusions were controversial, with the ‘obesity paradox’ being a notable issue. Some studies find that high BMI was closely associated with higher incidence and poorer prognosis of breast cancer, which may be related to the high disease burden caused by obesity [5,6]. Other studies suggest that low BMI in breast cancer patients was associated with lower survival rates and worse outcomes, possibly due to reduced muscle mass or malnutrition [7,8]. Additionally, a few studies argue that BMI has no impact on survival outcomes [9].

BMI significantly influenced the efficacy of anti-tumour strategies, but there are fewer studies on the relationship between BMI and the therapeutic effects of CDK4/6 inhibitors, and existing research generally suggests that BMI was not associated with the prognosis of CDK4/6 inhibitor therapy. In studies targeting HR-positive early breast cancer, the results of the PALLAS trial indicate that BMI has no significant correlation with the survival of patients without invasive disease. However, high BMI was associated with a reduced incidence of adverse reactions related to CDK4/6 inhibitors [10]. Regarding the use of CDK4/6 inhibitors in the treatment of HR-positive advanced breast cancer, multiple studies from the United States, the Netherlands, and Italy have shown that BMI has no significant association with overall survival (OS) and progression-free survival (PFS) [11–13]. However, a real-world study in an Asian population found that the risk of recurrence was very similar across different BMI, but the difference remained statistically significant (HR = 0.943, *p* = 0.003) [14]. Therefore, the exact relationship between BMI and the therapeutic effects of CDK4/6 inhibitors still requires further exploration.

Therefore, identifying prognostic factors for CDK4/6 inhibitor efficacy was critical to optimize individualized treatment strategies in HR-positive advanced breast cancer. Moreover, current research conclusions on the impact of BMI on the efficacy of CDK4/6 inhibitor therapy in HR-positive advanced breast cancer were inconsistent. This study retrospectively analysed clinical data from patients with metastatic HR-positive breast cancer treated with CDK4/6 inhibitors, revealing that a high BMI was linked to poorer overall survival outcomes following CDK4/6 inhibitor therapy.

## Methods

### Patient population

This study included all female patients with metastatic HR-positive breast cancer on a CDK4/6 inhibitor (including Palbociclib, Ribociclib, Abemaciclib or Dalpiciclib) therapy, seen in the first ward of the Department of Breast Medicine, Liaoning Cancer Hospital between July 2016 and November 2023. Inclusion criteria were as follows: (1) diagnosis of HR-positive breast cancer, (2) first- or late-line treatment with a CDK4/6 inhibitor, (3) complete clinicopathological data, and (4) female. All patients in this study were HR-positive as defined by immunohistochemistry with estimated percentages of nuclei staining of oestrogen receptor (ER) and/or progesterone receptor (PR) protein ≥ 10%. HER2-High status was defined by IHC 3+ or 2+ with positive ISH. HER2-Low status was defined by IHC 1+ or 2+ with negative ISH, and HER2-zero by IHC 0. Patients without diagnostic and treatment data or those lost to follow-up were excluded. The ethics committee of Liaoning Cancer Hospital approved the study (KY20240325).

### Data collection

We conducted a retrospective analysis to extract demographic, BMI, clinicopathological, and tumour treatment information from the included patients. The primary endpoint was OS, defined as the time from the start of CDK4/6 inhibitors to death due to any cause. The secondary endpoint was PFS, defined as the time from the start of CDK4/6 inhibitor therapy to disease progression or death due to any cause. Patients without OS events were censored on the last day of using CDK4/6 inhibitor. Follow-up occurred until March 31, 2025.

In the context of Chinese individuals, the BMI-low group is defined as having a BMI below 24, while the BMI-high group is defined as having a BMI equal to or over 24 kg/m^2^ [15]. Each patient’s BMI was calculated based on the measurement recorded at baseline when they began CDK4/6 inhibitor therapy. Endocrine drugs used in the same line with a CDK4/6 inhibitor were considered partners, including SERMs (Tamoxifen or Toremifen), AI (Letrozole, Anastrozole, or Exemestane), and SERDs (Fulvestrant).

### Statistical analysis

The SPSS 26 version was used for analysing each parameter. Qualitative data were typically presented in the form of patient numbers (*n*) and related percentages (%), while quantitative data were given based on normal distribution, either as mean ± standard deviation (x ± s) or median (including the 25th and 75th percentiles). Comparative analyses were conducted using the Fisher exact test and the Pearson chi-square test. The OS and PFS between groups were compared using the Kaplan–Meier curve and log-rank test. Both univariate and multivariate analyses employed the Cox proportional hazards regression model. All *P*-values were two-sided, and *P*-values less than 0.05 were considered statistically significant.

## Results

### Patient population

This study included 212 HR-positive advanced breast cancer patients who received CDK4/6 therapy at our department. The baseline demographic and clinical characteristics of patients are shown in Table 1. The median age was 57 years, with 90.6% of patients identified as postmenopausal. 95.8% of patients received CDK4/6 inhibitors in combination with endocrine therapy. Overall, 27.7% of patients received Palbociclib treatment, 12.2% received Ribociclib treatment, 32.9% received Abemaciclib treatment, and 27.8% received Dalpiciclib treatment. Among the 212 patients, 53.3% (113) were categorized as BMI-low (defined as BMI <24 kg/m^2^), and 46.7% (99) were categorized as BMI-high (defined as BMI ≥ 24 kg/m^2^). There were no statistically significant differences between the two groups in terms of age, menopausal status, prior medical history, tumour biomarker levels, or treatment regimens.

**Table 1.** Patient demographics and clinical characteristics.

Characteristic	Total (*N* = 212)	BMI < 24 *N* = 113 (7%)	BMI ≥ 24 *N* = 99 (10.8%)	*p*
Age (years)	57.18 ± 11.15	57.11 ± 11.08	57.26 ± 11.29	0.919
Menopause status (n, %)				0.873
Yes	192 (90.6%)	102 (90.3%)	90 (90.9%)	
No	20 (9.4%)	11 (9.7%)	9 (9.1%)	
Hypertension (n, %)				0.707
Yes	28 (13.2%)	14 (12.4%)	14 (14.1%)	
No	184 (86.8%)	99 (87.6%)	85 (85.9%)	
Diabetes (n, %)				0.418
Yes	14 (6.6%)	6 (5.3%)	7 (8.1%)	
No	198 (93.4%)	107 (94.7%)	91 (91.9%)	
Ischaemic heart disease (n, %)				0.278
Yes	10 (4.7%)	7 (6.2%)	3 (3.0%)	
No	202 (95.3%)	106 (93.8%)	96 (97.0%)	
Smoking (n, %)				0.894
Yes	4 (1.9%)	2 (1.8%)	2 (2.0%)	
No	208 (98.1%)	111 (98.2%)	97 (98.0%)	
Alcohol (n, %)				–
Yes	0 (0%)	0 (0%)	0 (0%)	
No	212 (100%)	113 (100%)	99 (100%)	
Carcino-embryonic antigen (ng/ml)	5.05 (2.34–12.86)	5.89 (2.84–18.13)	4.46 (2.09–7.42)	0.010
CA125 (U/mL)	17.20 (11.40–63.53)	17.40 (11.45–70.50)	17.20 (11.40–60.00)	0.789
CA153 (U/mL)	19.69 (8.27–46.73)	21.93 (7.86–81.89)	19.60 (9.59–32.30)	0.207
CDK4/6 inhibitors (n, %)				0.296
Palbociclib	59 (27.8%)	35 (31.0%)	24 (24.2%)	
Ribociclib	26 (12.3%)	10 (8.8%)	16 (16.2%)	
Abemaciclib	69 (32.5%)	39 (34.5%)	30 (30.3%)	
Dalpiciclib	58 (27.4%)	29 (25.7%)	29 (29.3%)	
Drug partner (n, %)				0.891
No partner	9 (4.2%)	4 (3.5%)	5 (5.1%)	
SERM	7 (3.3%)	4 (3.5%)	3 (3.0%)	
AI	116 (54.7%)	64 (56.6%)	52 (52.5%)	
SERD	80 (37.7%)	41 (36.3%)	39 (39.4%)	

### Tumour characteristics of patients

Tumour characteristics were shown in Table 2. No statistically significant differences were observed between the BMI-low and BMI-high groups in terms of surgical procedures and histological subtypes. There were also no statistically significant differences in the number of lines of tumour treatment or metastatic sites between the two groups. Compared to the BMI-low group, the BMI-high group did not show statistically significant differences in ER, PR, HER2, and Ki-67 expression.

**Table 2.** Tumour characteristics.

Characteristic	Total (*N* = 212)	BMI < 24 *N* = 113	BMI ≥ 24 *N* = 99	*χ^2^*	*P*
Surgery (n, %)				2.72	0.257
Modified radical mastectomy	122 (57.5%)	62 (54.9%)	60 (60.6%)		
Conservative surgery and others	40 (18.9%)	26 (23.0%)	14 (14.1%)		
No (Newly diagnosed advanced)	50 (23.6%)	25 (22.1%)	25 (25.3%)		
Histological Subtype (n, %)				0.844	0.656
Invasive ductal carcinoma	154 (72.6%)	82 (72.6%)	72 (72.7%)		
Invasive lobular carcinoma	12 (5.7%)	5 (4.4%)	7 (7.1%)		
Others	46 (21.7%)	26 (23.0%)	20 (20.2%)		
Line (n, %)				3.103	0.078
1	117 (55.2%)	56 (49.6%)	61 (61.1%)		
≥ 2	95 (44.8%)	57 (50.4%)	38 (38.4%)		
Bone metastases (n, %)				0.005	0.946
Yes	129 (60.8%)	69 (61.1%)	60 (60.6%)		
No	83 (39.2%)	44 (38.9%)	39 (39.4%)		
Liver metastases (n, %)				0.711	0.399
Yes	55 (25.9%)	32 (28.3%)	23 (23.2%)		
No	157 (74.1%)	81 (71.7%)	76 (76.8%)		
lung metastases (n, %)				0.758	0.384
Yes	77 (36.3%)	38 (33.6%)	39 (39.4%)		
No	135 (63.7%)	75 (66.4%)	60 (60.6%)		
Chest wall or others metastasis (n, %)				0.142	0.706
Yes	117 (55.2%)	61 (54.0%)	56 (56.6%)		
No	95 (44.8%)	52 (46.0%)	43 (43.4%)		
Oestrogen receptor status (n, %)				0.530	0.767
≤ 10%	5 (2.4%)	3 (2.7%)	2 (2.0%)		
≥ 10%, < 90%	91 (42.9%)	46 (40.7%)	45 (45.5%)		
≥ 90%	116 (54.7%)	64 (56.6%)	52 (52.5%)		
Progesterone receptor status (n, %)				5.373	0.068
≤ 10%	61 (28.8%)	39 (34.5%)	22 (22.2%)		
≥ 10%, < 90%	103 (48.6%)	47 (41.6%)	56 (56.6%)		
≥ 90%	48 (22.6%)	27 (23.9%)	21 (21.2%)		
HER-2 receptor status (n, %)				0.540	0.763
Negative	78 (36.8%)	41 (36.3%)	37 (37.4%)		
Low (1 or 2 + and fısh negative)	131 (61.8%)	71 (62.8%)	60 (60.6%)		
High (3 or 2 + and fısh positive)	3 (1.4%)	1 (0.9%)	2 (2.0%)		
Ki-67 status (n, %)				0.396	0.820
≤ 15%	57 (26.9%)	30 (26.5%)	27 (27.3%)		
≥ 15%, < 30%	38 (17.9%)	22 (19.5%)	16 (16.2%)		
≥ 30%	117 (55.2%)	61 (54.0%)	56 (56.6%)		

### OS and PFS

**Figure 1.**
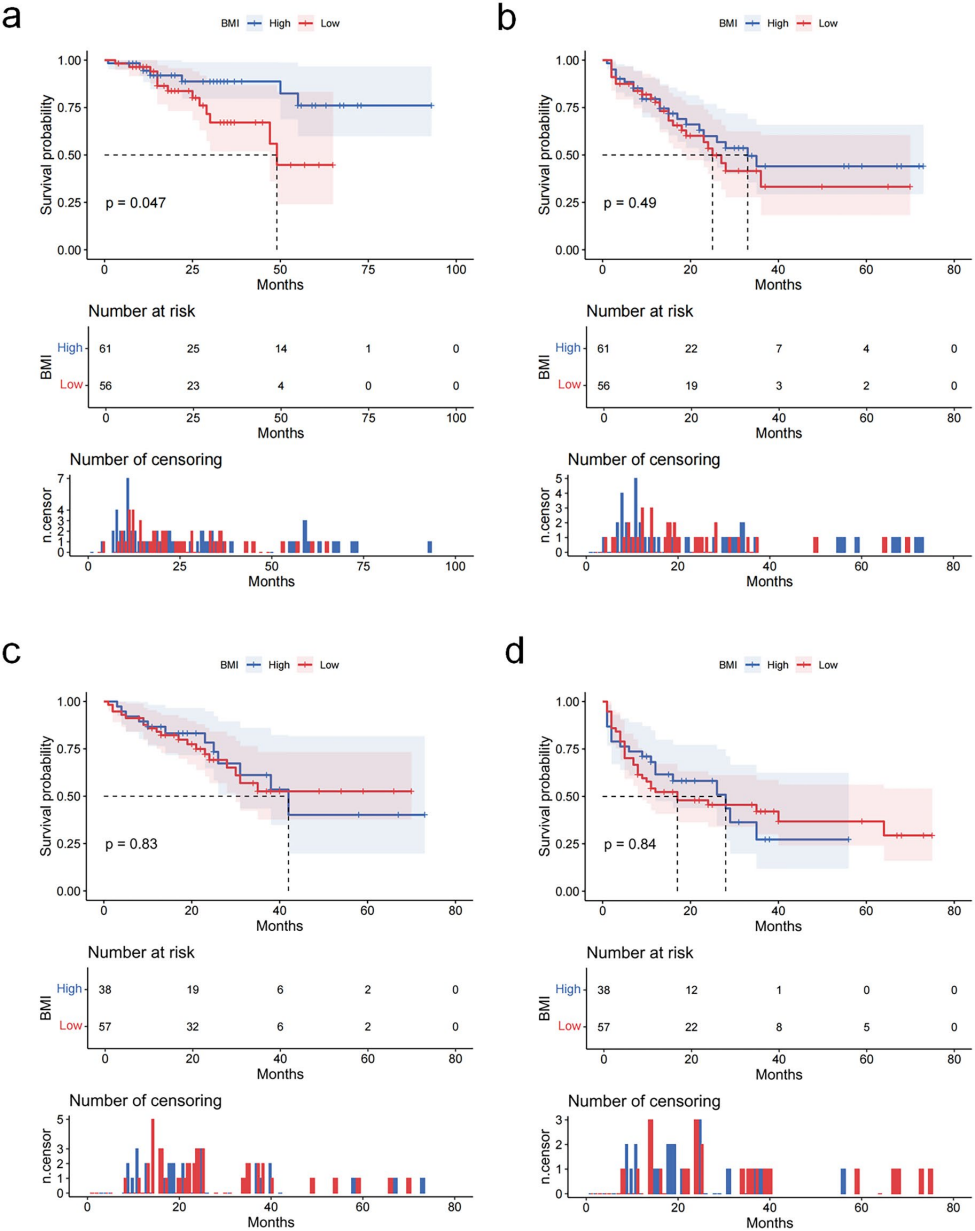
Overall survival and progression-free survival according to BMI. Survival curves were generated using Kaplan-Meier survival analysis. Between-group differences were assessed by log-rank tests. Data points represent median survival times (months) with 95% confidence intervals (CI). a. OS of first-line CDK4/6 inhibitors, *n* = 117, *p* = 0.047. b. PFS of first-line CDK4/6 inhibitors, *n* = 117, *p* = 0.49. c. OS of late-line CDK4/6 inhibitors, *n* = 95, *p* = 0.83. d. PFS of late-line CDK4/6 inhibitors, *n* = 95, *p* = 0.84.

In 212 HR-positive advanced breast cancer patients treated with CDK4/6, the median follow-up time was 20 months. During the follow-up period, 51 patients died, including 32 in the BMI-low group and 19 in the BMI-high group. In the first-line data analysis, the median overall survival for the BMI-High group had not been reached. The mean OS time for BMI-low group was 46.6 months (95% CI, 38.9 to 54.3), and for the BMI-high group, it was 78.9 months (95% CI, 69.2 to 88.5) (Figure 1(a)). This difference was determined to be statistically significant (*p* = 0.047). The mPFS for the BMI-low group was 25 months (95% CI, 19.7 to 30.3), and for the BMI-high group, it was 33 months (95% CI, 21.4 to 44.6) (Figure 1(b)). Patients with higher BMI exhibited longer PFS and improved outcomes compared to those with lower BMI. It was worth noting that this difference did not reach statistical significance (*p* = 0.488). In the late-line analysis of CDK4/6 treatment, the median overall survival for the BMI-low group had not been reached. For the BMI-low group, the mean OS time was 45.9 months (95% CI, 37.5 to 54.3), and for the BMI-high group, it was 45.0 months (95% CI, 33.2 to 56.7) (Figure 1(c)). The mPFS was 17 months (95% CI 10.8 to 38.1) in the BMI-low group and 28 months (95% CI, 10.4 to 45.6) in the BMI-high group (Figure 1(d)). No statistically significant difference was observed between OS and PFS at the late line (*p* = 0.83, *p* = 0.84).

Differences in OS and PFS between BMI-low and BMI-high groups for first-line and late-line CDK4/6 inhibitors and drug partner subgroups were detailed in Supplementary 1 (Tables S1–S2, Figures S1–S4). Compared to the BMI-low group, a significantly longer OS was observed exclusively among patients receiving first-line CDK4/6 inhibitors combined with AI (*p* = 0.002) (Figure S2). No statistically significant differences were observed in other subgroups (Figures S1–S4).

### Prognostic factors of OS treated with first-line CDK4/6 inhibitors

Tables 3 and 4 summarized the univariate and multivariate analyses of OS associated with first-line CDK4/6 inhibitor therapy. In the univariate analysis, BMI (*p* = 0.018), carcino-embryonic antigen (*p* = 0.049), CA125 (*p* < 0.001), CA153 (*p* = 0.011), and liver metastasis (*p* = 0.014) were all associated with OS. Although carcino-embryonic antigen, CA125, and CA153 showed statistical significance in univariate analysis, their HR were close to 1 (carcino-embryonic antigen: HR = 1.001; CA125: HR = 1.003; CA153: HR = 1.004), indicating negligible clinical impact on outcomes. To avoid overfitting and maintain model parsimony, these biomarkers were excluded from the multivariate analysis. Based on the multivariate analysis, poor OS in first-line CDK4/6 inhibitor therapy was independently associated with low BMI (*p* = 0.023) and liver metastasis (*p* = 0.017).

**Table 3.** Univariate analysis of factors affecting OS of first-line CDK4/6 inhibitors.

Characteristic	Number *N* = 117	Univariate analysis		
		HR	95%CI	*p* value
Age (years)	56.93 ± 10.64	0.997	0.954, 1.043	0.906
BMI (kg/m^2^)	23.93 ± 3.74	0.861	0.761, 0.975	0.018
Menopause status (n, %)				
Yes	106 (90.6%)	0.425	0.123, 1.468S	0.176
No	11 (9.4%)	–	–	
Hypertension (n, %)				
Yes	16 (13.7%)	2.569	0.847, 7.797	0.096
No	101 (86.3%)	–	–	
Carcino-embryonic antigen (ng/ml)	4.46 (1.90–8.86)	1.001	1.000, 1.002	0.049
CA125 (U/mL)	15.90 (10.60–55.70)	1.003	1.001, 1.005	< 0.001
CA153 (U/mL)	17.71 (6.98–32.60)	1.004	1.001, 1.007	0.011
CDK4/6 inhibitors (n, %)		0.687	0.468	0.056
Palbociclib	25 (21.4%)	–	–	
Ribociclib	16 (13.7%)	–	–	
Abemaciclib	38 (32.5%)	–	–	
Dalpiciclib	38 (32.5%)	–	–	
Drug partner (n, %)		1.470	0.732, 2.952	0.279
No partner	6 (5.1%)	–	–	
SERM	3 (2.6%)	–	–	
AI	77 (65.8%)	–	–	
SERD	31 (26.5%)	–	–	
Surgery (n, %)		0.972	0.497, 1.898	0.933
Modified radical mastectomy	63 (53.8%)	–	–	
Conservative surgery and others	20 (17.1%)	–	–	
No (Newly diagnosed advanced)	34 (29.1%)	–	–	
Histological Subtype (n, %)		0.950	0.575, 1.570	0.841
Invasive ductal carcinoma	80 (68.4%)	–	–	
Invasive lobular carcinoma	7 (6.0%)	–	–	
Others	30 (25.6%)	–	–	
Bone metastases (n, %)				
Yes	66 (56.4%)	1.582	0.607, 4.127	0.348
No	51 (43.6%)	–	–	
Liver metastases (n, %)				
Yes	22 (18.8%)	3.081	1.257, 7.553	0.014
No	95 (81.2%)	–	–	
lung metastases (n, %)				
Yes	37 (1.6%)	0.830	0.318, 2.167	0.704
No	80 (68.4%)	–	–	
Chest wall or others metastasis (n, %)				
Yes	64 (54.7%)	1.580	0.644, 3.875	0.318
No	53 (45.3%)	–	–	
Oestrogen receptor status (n, %)		0.791	0.370, 1.692	0.546
≤ 10%	3 (2.5%)	–	–	
≥ 10%, < 90%	45 (38.5%)	–	–	
≥ 90%	69 (59.0%)	–	–	
Progesterone receptor status (n, %)		1.070	0.531, 2.157	0.850
≤ 10%	28 (23.9%)	–	–	
≥ 10%, < 90%	63 (53.8%)	–	–	
≥ 90%	26 (22.2%)	–	–	
HER-2 receptor status (n, %)		0.716	0.301, 1.706	0.451
Negative	41 (35.0%)	–	–	
Low (1 or 2 + and fısh negative)	75 (64.1%)	–	–	
High (3 or 2 + and fısh positive)	1 (0.9%)	–	–	
Ki-67 status (n, %)		1.106	0.664, 1.843	0.699
≤ 15%	32 (27.4%)	–	–	
≥ 15%, < 30%	22 (18.8%)	–	–	
≥ 30%	63 (53.8%)	–	–	

**Table 4.** Multivariate analysis of factors affecting OS of first-line CDK4/6 inhibitors.

Characteristic	Number *N* = 117	Univariate analysis		
		HR	95%CI	*p* value
BMI (kg/m^2^)	23.93 ± 3.74	0.865	0.763, 0.980	0.023
Liver metastases (n, %)				
Yes	22 (18.8%)	2.994	1.219, 7.352	0.017
No	95 (81.2%)	–	–	

## Discussion

The primary aim of this study was to evaluate the impact of BMI on the efficacy of CDK4/6 inhibitors in patients with metastatic HR-positive breast cancer. This study used China’s obesity standards to classify patients into two groups based on BMI. We found that in the BMI-high group of HR-positive advanced breast cancer patients treated with first-line CDK4/6 inhibitors, OS was significantly longer than in the BMI-low group (46.6 vs. 78.9 months, *p* = 0.047), and there was also a trend toward extended PFS (25 vs. 33 months), although the latter did not reach statistical significance. Furthermore, the absence of a significant association between BMI and late-line survival outcomes may be attributed to potential constraints in the current study, including limited sample size and follow-up duration. In summary, these findings challenged traditional views, suggesting that high BMI may confer protective effects in CDK4/6 inhibitor therapy.

Our research findings did not align with the traditional conclusions. Generally, obesity was considered a significant risk factor for the development, progression, and recurrence of breast cancer [16–18]. In studies examining obesity-related mechanisms of cancer, conclusions usually suggested that obesity might drive cancer growth, invasion, and metastasis by increasing local and circulating pro-inflammatory cytokines, promoting tumour angiogenesis, and stimulating cancer stem cell populations [19]. Additionally, high BMI is often associated with hyperglycaemia, which might be a predictor of poor recurrence-free survival in HR-positive breast cancer patients [20]. Moreover, HR-positive breast cancer patients with high BMI have a higher risk of developing non-alcoholic fatty liver disease, and those with this condition have poor disease-free survival compared to those without [21]. Furthermore, postmenopausal women with low BMI exhibited higher cancer type recurrence scores [22]. In studies on endocrine therapy, it has also been found that compared to normal-weight patients, high-BMI patients have significantly poor breast cancer-specific survival [23].

However, in recent years, the ‘obesity paradox’ theory has emerged, suggesting that patients with higher BMIs have a lower risk of breast cancer, as well as a lower risk of disease progression and recurrence [24]. This implied that high BMI may act as a protective factor against breast cancer, while low BMI becomes a risk factor. Molecular mechanism research in postmenopausal breast cancer populations has revealed that the “obesity paradox” may be associated with the PI3K-AKT signalling pathway, proteoglycans in cancer, and pathways related to lipid metabolism and atherosclerosis [25]. Furthermore, the gut microbiome and tumour microbiome may modulate therapeutic efficacy and drug resistance in breast cancer by influencing lipid metabolism and the immune microenvironment [26–28]. Research in melanoma has suggested that the “obesity paradox” might arise from obesity-induced alterations in gut microbiome composition, thereby impacting the efficacy of targeted therapy and immunotherapy [29]. Studies suggested that poor prognosis associated with low BMI may be related to insufficient muscle mass and malnutrition [30]. The study of triple-negative breast cancer in China found that sarcopenia was associated with poorer clinical outcomes [8]. Additionally, the multicentre analysis using individual data from 758,592 premenopausal women across 19 prospective cohorts found a negative linear association between BMI and breast cancer risk [31]. Further study indicated that this phenomenon may be age-dependent. Specifically, higher BMI was associated with less breast cancer incidence in females younger than 55 years of age (OR = 0.313, CI: 0.240–0.407) [32]. Turkish studies have shown that among metastatic breast cancer patients treated with CDK4/6 inhibitors, low BMI is associated with poor PFS, whereas overweight patients have a longer PFS (9.3 vs. 11.1 months, *p* = 0.02) [33]. Similarly, a study involving 159,248 patients in the United States found that compared to high-BMI groups, low-BMI breast cancer patients had early diagnosis but poor specific survival [34]. Our research findings are consistent with these conclusions.

The principal limitations of this study include its modest sample size and single-center, single-race design, which may introduce potential biases. Additionally, the present study did not assess potential confounding variables, including performance status, CDK4/6 inhibitor dose adjustments, and metabolic comorbidities such as dysregulation of fatty acids, cholesterol, insulin, and leptin, which may be associated with BMI-related impact on the efficacy of CDK4/6 inhibitors. Notably, prospective studies have shown that even within the normal range of BMI, an increase of 5 kg in trunk fat can raise the risk of ER-positive breast cancer by 5% [35]. Therefore, future research should consider the impact of abnormal body fat on the efficacy of CDK4/6 inhibitors in individuals with normal BMI.

## Conclusion

In this study, we evaluated treatment data from a group of metastatic HR-positive breast cancer patients treated with CDK4/6 inhibitors at a single center and reported factors influencing poor prognosis. Among patients receiving first-line CDK4/6 inhibitors, there were differences in outcomes across different BMI groups. We found that low BMI and liver metastasis were adverse prognostic variables affecting OS. Future research may leverage these findings to inform personalized treatment strategies for metastatic HR-positive breast cancer patients.

## Supplementary Material

Supplemental Material

## Data Availability

The data that support the findings of this study are available from the corresponding author upon reasonable request.
