# Supplementary material for: The impact of body mass index on the efficacy of CDK4/6 inhibitors in patients with metastatic breast cancer
Source: Ann Med. 2025 Dec 4;57(1):2597068. doi: 10.1080/07853890.2025.2597068 (PMC12683747; doi:10.1080/07853890.2025.2597068)
Supplement: Supplemental Material [file IANN_A_2597068_SM4815.zip › Supplemental/Table S1.docx]

Table S1 First-line patient CDK4/6 inhibitors and drug partner characteristics

| **Characteristic** | **Total**  **(*N*=117)** | **BMI < 24**  ***N* = 56 (47.9%)** | **BMI ≥ 24**  ***N* = 61 (52.1%)** | ***p*** |
| --- | --- | --- | --- | --- |
| CDK4/6 inhibitors (n, %) |  |  |  | 0.474 |
| Palbociclib | 25 (21.4%) | 14 (25.0%) | 11 (18.0%) |  |
| Ribociclib | 16 (13.7%) | 5 (8.9%) | 11 (18.0%) |  |
| Abemaciclib | 38 (32.5%) | 19 (33.9%) | 19 (31.1%) |  |
| Dalpiciclib | 38 (32.5%) | 18 (32.1%) | 20 (32.8%) |  |
| Drug partner (n, %) |  |  |  | 0.477 |
| No partner | 6 (5.1%) | 2 (3.6%) | 4 (6.6%) |  |
| SERM | 3 (2.6%) | 2 (3.6%) | 1 (1.6%) |  |
| AI | 77 (65.8%) | 40 (71.4%) | 37 (60.7%) |  |
| SERD | 31 (26.5%) | 12 (21.4%) | 19 (31.1%) |  |
