# Supplementary material for: The impact of body mass index on the efficacy of CDK4/6 inhibitors in patients with metastatic breast cancer
Source: Ann Med. 2025 Dec 4;57(1):2597068. doi: 10.1080/07853890.2025.2597068 (PMC12683747; doi:10.1080/07853890.2025.2597068)
Supplement: Supplemental Material [file IANN_A_2597068_SM4815.zip › Supplemental/Table S2.docx]

Table S2 Late-line patient CDK4/6 inhibitors and drug partner characteristics

| **Characteristic** | **Total**  **(*N*=95)** | **BMI < 24**  ***N* = 57 (60%)** | **BMI ≥ 24**  ***N* = 38 (40%)** | ***p*** |
| --- | --- | --- | --- | --- |
| CDK4/6 inhibitors (n, %) |  |  |  | 0.818 |
| Palbociclib | 34 (35.8%) | 21 (36.8%) | 13 (34.2%) |  |
| Ribociclib | 10 (10.5%) | 5 (8.8%) | 5 (13.2%) |  |
| Abemaciclib | 31 (32.6%) | 20 (35.1%) | 11 (28.9%) |  |
| Dalpiciclib | 20 (21.1%) | 11 (19.3%) | 9 (23.7%) |  |
| Drug partner (n, %) |  |  |  | 0.965 |
| No partner | 3 (3.2%) | 2 (3.5%) | 1 (2.6%) |  |
| SERM | 4 (4.2%) | 2 (3.5%) | 2 (5.3%) |  |
| AI | 39 (41.1%) | 24 (42.1%) | 15 (39.5%) |  |
| SERD | 49 (51.6%) | 29 (50.9%) | 20 (52.6%) |  |
