# Supplementary figures and images for: The impact of body mass index on the efficacy of CDK4/6 inhibitors in patients with metastatic breast cancer
Source: Ann Med. 2025 Dec 4;57(1):2597068. doi: 10.1080/07853890.2025.2597068 (PMC12683747; doi:10.1080/07853890.2025.2597068)

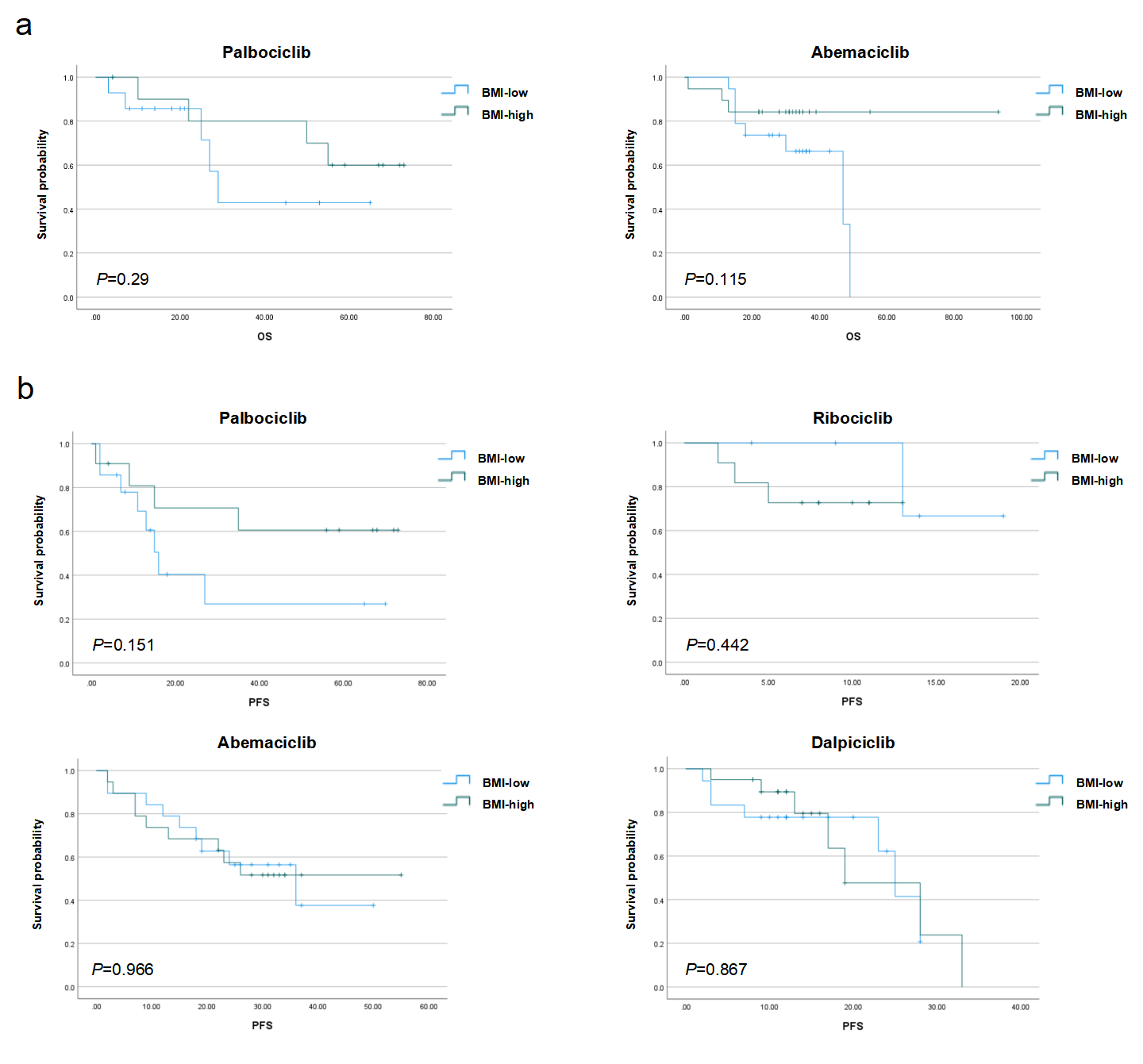

Supplement: Supplemental Material [file IANN_A_2597068_SM4815.zip › Supplemental/Figure S1.tif]

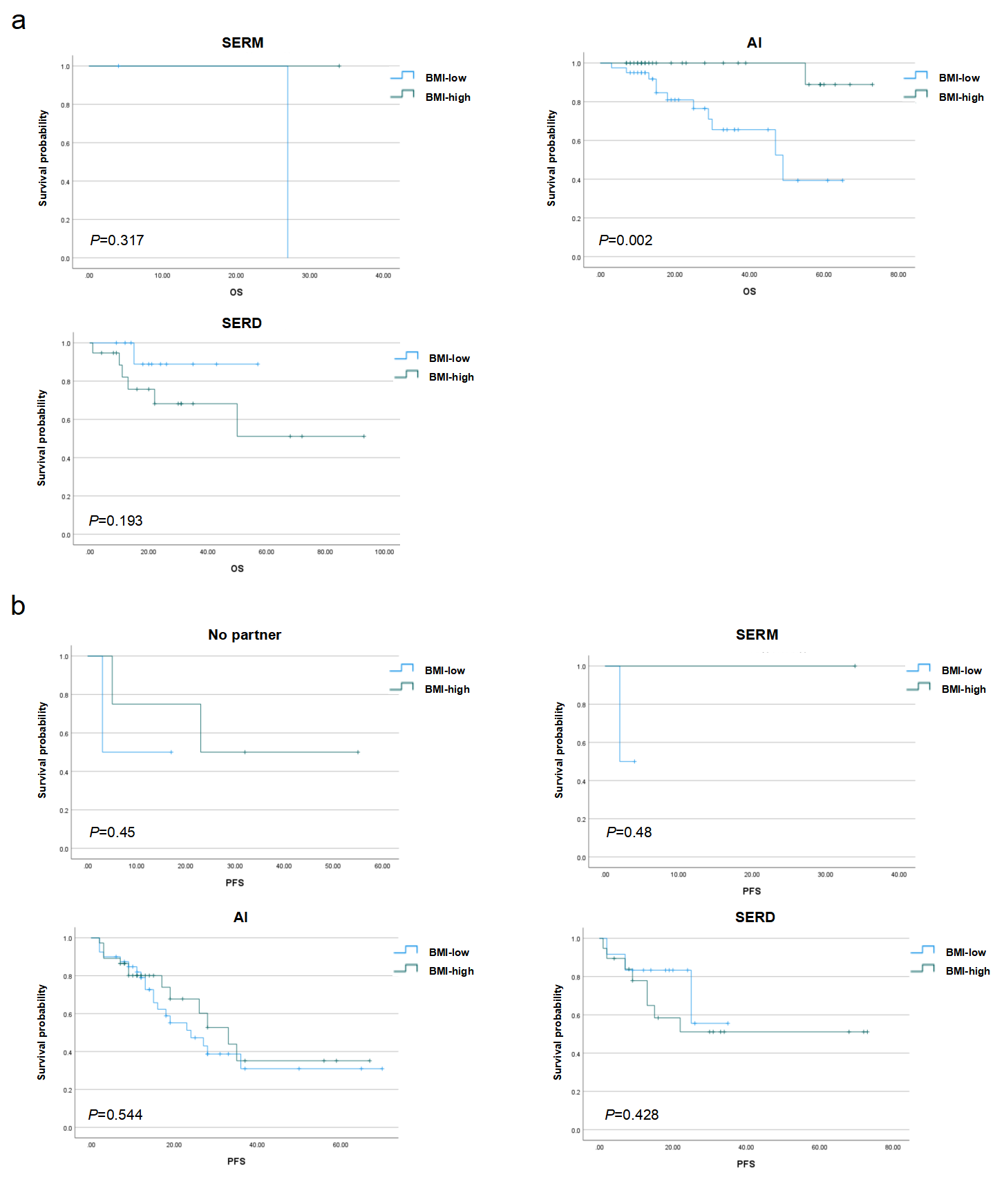

Supplement: Supplemental Material [file IANN_A_2597068_SM4815.zip › Supplemental/Figure S2.tif]

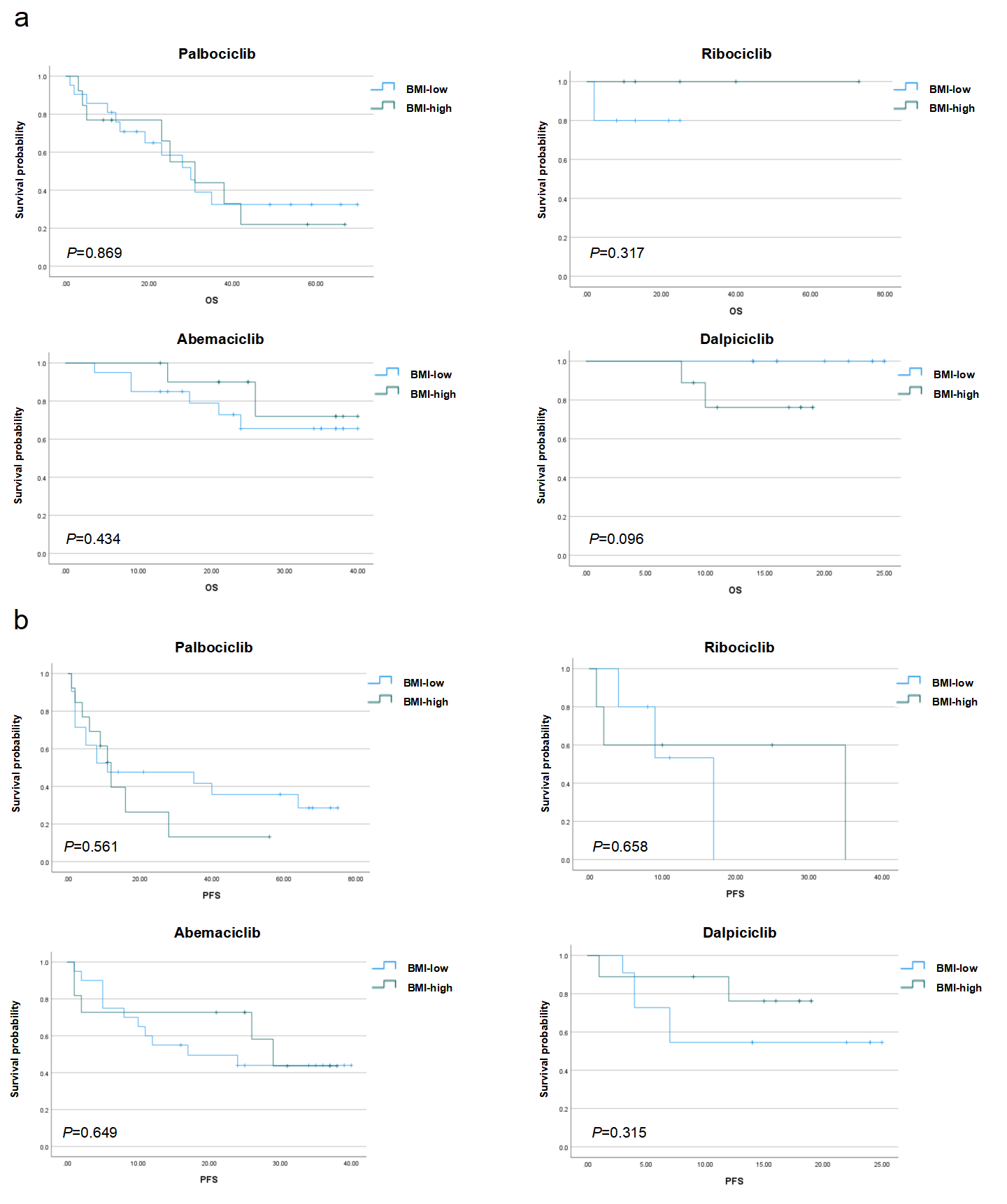

Supplement: Supplemental Material [file IANN_A_2597068_SM4815.zip › Supplemental/Figure S3.tif]

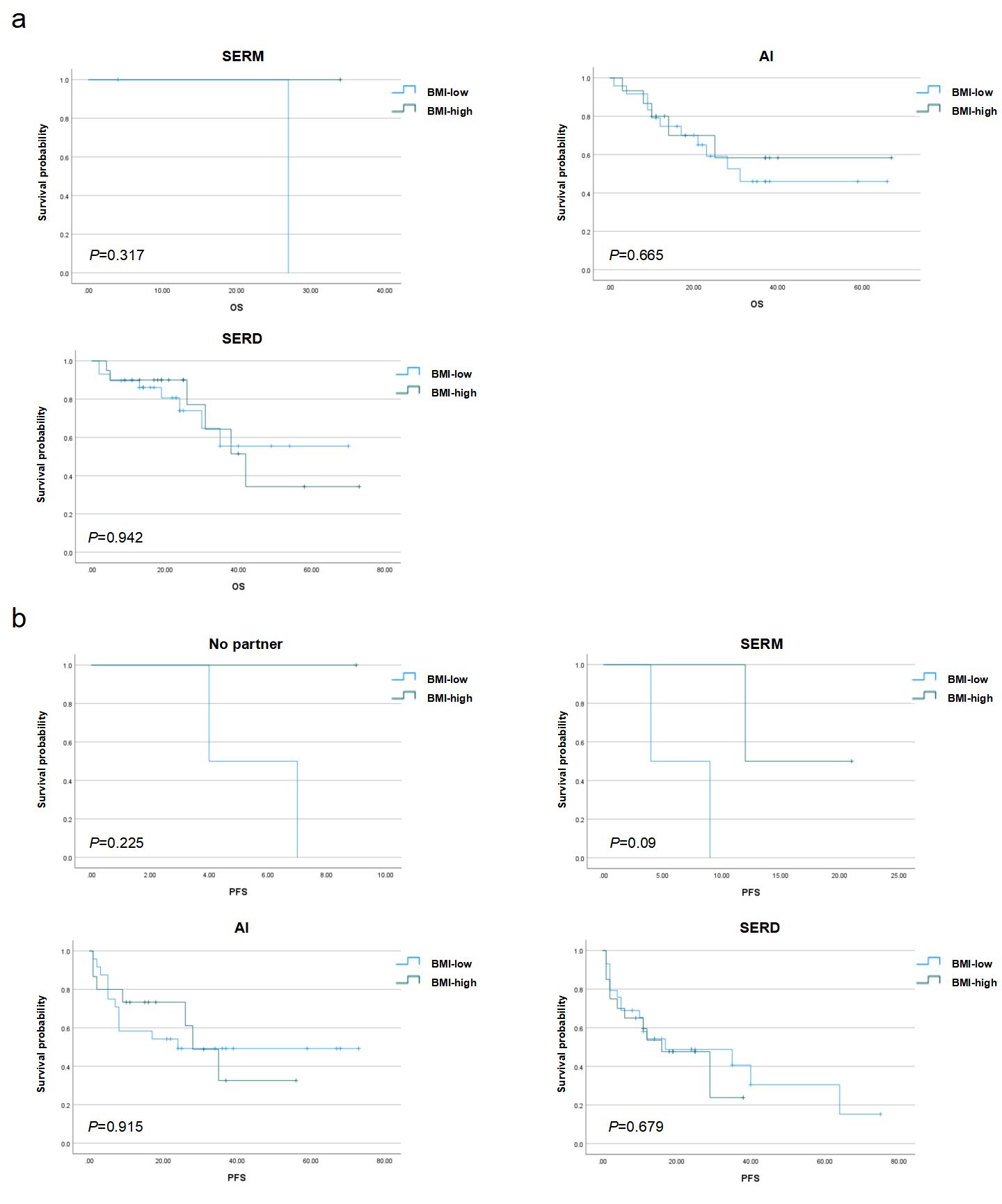

Supplement: Supplemental Material [file IANN_A_2597068_SM4815.zip › Supplemental/Figure S4.tif]
